# Expansion of Invasive Group A *Streptococcus* M1_UK_ Lineage in Active Bacterial Core Surveillance, United States, 2019‒2021

**DOI:** 10.3201/eid2910.230675

**Published:** 2023-10

**Authors:** Yuan Li, Joy Rivers, Saundra Mathis, Zhongya Li, Sopio Chochua, Benjamin J. Metcalf, Bernard Beall, Jennifer Onukwube, Christopher J. Gregory, Lesley McGee

**Affiliations:** Centers for Disease Control and Prevention, Atlanta, Georgia, USA

**Keywords:** invasive group A Streptococcus, streptococci, bacteria, invasive disease, iGAS, M1_UK_ lineage, clonal expansion, respiratory infections, zoonoses

## Abstract

From 2015–2018 to 2019‒2021, hypertoxigenic M1_UK_ lineage among invasive group A *Streptococcus* increased in the United States (1.7%, 21/1,230 to 11%, 65/603; p<0.001). M1_UK_ was observed in 9 of 10 states, concentrated in Georgia (n = 41), Tennessee (n = 13), and New York (n = 13). Genomic cluster analysis indicated recent expansions.

The M1_UK_ lineage of group A *Streptococcus* (GAS) is a hypertoxigenic clone within the serotype M1 GAS strain and has been associated with increased scarlet fever and invasive GAS (iGAS) disease incidence in the United Kingdom since 2014 (*1**–**3*). M1_UK_ carries 27 characteristic lineage-defining single-nucleotide variants (SNVs) that distinguish it from other globally circulating M1 GAS clones (*1*). By 2020, M1_UK_ had also became the dominant clone among M1 GAS in England (*3*), the Netherlands (*4*), and Australia (*5*) and showed substantial presence in Canada (*6*).

In the United States, M1_UK_ was identified as a minor clone of M1 iGAS isolates in the Active Bacterial Core surveillance (ABCs) system, a laboratory- and population-based surveillance system for invasive bacterial infections that is currently implemented in 10 US states (*7*), in 2015–2018 (*8*). Using genomic surveillance data in ABCs, we investigated the trend of M1_UK_ in 2019–2021 and documented the characteristics of iGAS infections caused by M1_UK_.

## The Study

We identified iGAS cases through ABCs and mapped whole-genome sequencing reads of M1 isolates against the M1 reference genome MGAS5005 to identify M1_UK_ based on previously reported characteristic M1_UK_ SNVs (*1*). We constructed phylogenetic trees by using kSNP3.0 software (*9*). We identified genomic clusters by using a hierarchical cluster analysis with a cutoff value of 10 SNVs as described (*10*). We evaluated change of M1_UK_ proportion among M1 iGAS over time by using the χ^2^ test for trend in proportions (trend test). We used the Fisher exact test to assess equality of proportions. All p values were 2 sided, and we considered p<0.05 statistically significant. We performed all analyses by using R software version 3.4.3 (The R Foundation for Statistical Computing, https://www.r-project.org).

We submitted all whole-genome sequencing data files of the study isolates to the National Center for Biotechnology Information Sequence Read Archive (BioProject no. PRJNA395240). Accession numbers of the 86 M1_UK_ isolates are provided (Appendix Table 4).

**Figure 1:**
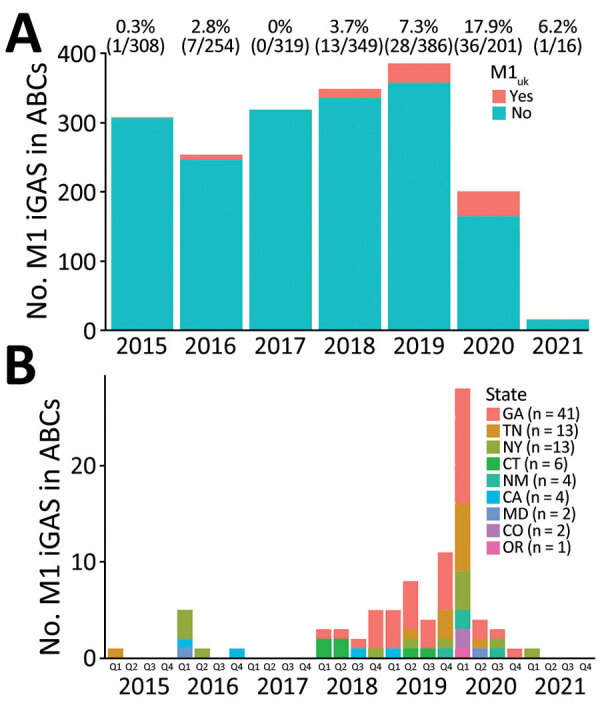
Expansion of M1_UK_ lineage in serotype M1 iGAS in the United States, 2015–2021. A) Counts and percentages of M1_UK_ isolates among M1 iGAS isolates in ABCs during 2015‒2021. B) Number of M1_UK_ infections over time in 9 states that are part of the ABCs system. Key shows total number of M1_UK_ infections during 2015‒2021 for each state. ABCs, Active Bacterial Core Surveillance System; iGAS, invasive group A *Streptococcus* disease; Q, quarter.

During 2019‒2021, a total of 603 cases of M1 iGAS infections were documented through ABCs. Among those cases, 65 (11%) were caused by the M1_UK_ clone (Figure 1, panel A), and the percentage was significantly higher than that observed during 2015–2018 (1.7%, 21/1,230; p<0.001). The trend test indicated a significant increasing trend in the M1_UK_ proportion among M1 iGAS isolates during 2015‒2021 (p<0.001). During 2015–2021, most M1_UK_ cases (67/86) were concentrated in 3 states: Georgia (41 cases,) Tennessee (13 cases), and New York (13 cases), although the M1_UK_ clone was found in 9 of the 10 ABCs sites (Figure 1, panel B). Nearly one third of all M1_UK_ infections (28/86) occurred in the first quarter of 2020 (Figure 1, panel B). During 2015–2021, a total of 12 iGAS isolates were identified as the intermediate lineages, containing 13 (n = 4) or 23 (n = 8) of the 27 characteristic M1_UK_ SNVs (Appendix Figure 1), and they did not show significant expansion from 2015–2018 through 2019–2021 (p = 0.07).

**Figure 2:**
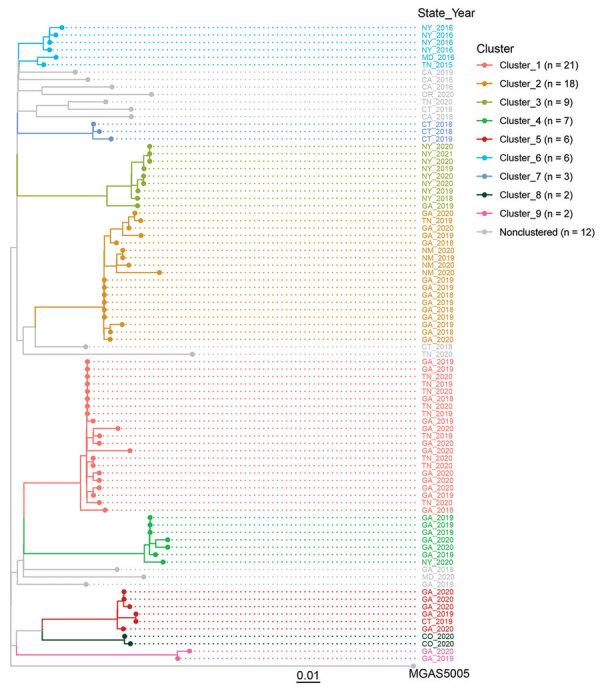
Genomic clusters of M1_UK_ invasive group A *Streptococcus* disease, United States, 2015–2021. Core-genome phylogenetic tree of 86 M1_UK_ invasive group A *Streptococcus* disease isolates and the reference M1 genome MGAS5005 was based on 462 core single-nucleotide variant sites generated by kSNP3.0 software (*9*). Tip colors indicate 9 groups of genomically closely related isolates (genomic clusters). Key shows total number of M1_UK_ isolates in each cluster. Scale bar indicates expected nucleotide substitutions per site.

Phylogenetic analysis of the 86 M1_UK_ isolates showed 9 distinctive genomic clusters (Figure 2). Each genomic cluster contained 2‒21 genomically closely related isolates, and collectively those clusters accounted for 74 (86%) of all M1_UK_ isolates (Figure 2). For 2 M1_UK_ isolates within a same cluster, the median pairwise genomic distance was 3 SNVs (interquartile range [IQR] 1.5‒6), consistent with continued transmission from a recent introduction event. However, for 2 M1_UK_ isolates not in the same cluster, the median pairwise genomic distance was 33 SNVs (IQR 28‒38), indicating some degree of genomic diversity within M1_UK_, although not as much as the diversity observed among 100 randomly selected globally circulating M1 GAS clone isolates in ABCs, 2015–2021 (median 63 SNVs, IQR 49‒115). (Appendix Figure 1).

The clusters displayed clear signatures of temporal and geographic relatedness (Appendix Figure 2). For example, the largest cluster, cluster_1, showed a sharp increase of cases at the beginning of 2020, followed by a rapid decrease. The second largest cluster, cluster 2, showed relatively stable case numbers spanning from the third quarter of 2018 to the third quarter of 2020. Within a genomic cluster, most M1_UK_ iGAS were identified in 1 or 2 states, suggesting a localized spread of the infection.

Overall, M1_UK_ and non-M1_UK_ M1 isolates had many common genetic features of the contemporary M1 *S. pyogenes* strain (Table). The M1_UK_ clone had a higher proportion of isolates that had the streptococcal pyrogenic exotoxin gene *speC* (4.7% [4/86] vs. 1.4% [24/1,747]; p = 0.039), the super antigen A gene *ssa* (2.3% [2/86] vs. 0.1% (2/1,747]; p = 0.012), and the extracellular streptodornase D gene *sda1* (also known as *sdaD2*; 100% [86/86] vs. 92% [1,613/1,747]; p = 0.002). The *speC* and *ssa* genes were found in 4 nonclustered M1_UK_ isolates, of which 2 isolates had both genes, suggesting acquisition of prophage ΦHKU488.vir (*5*).

**Table:** Strain and patient features of M1_UK_ iGAS compared with other M1 iGAS in ABCs, United States, 2015–2021

Characteristic	M1 iGAS, no. (%) cases		p value†
	M1_UK_, n = 86	Non-M1_UK_, n = 1,747	
Strain feature‡			
Antimicrobial susceptibility			
Penicillin nonsusceptible	0	0	1.000
Erythromycin nonsusceptible	0	21 (1.2)	0.621
Clindamycin nonsusceptible	0	20 (1.1)	1.000
Tetracycline nonsusceptible	0	18 (1)	1.000
Levofloxacin nonsusceptible	0	9 (0.5)	1.000
Pyrogenic exotoxin genes			
* speA*	86 (100)	1,720 (98.5)	0.635
* speC*	4 (4.7)	24 (1.4)	0.039
* speG*	86 (100)	1747 (100)	1.000
* speH*	0	1 (0.1)	1.000
* speI*	0	1 (0.1)	1.000
* speJ*	86 (100)	1,747 (100)	1.000
* speK*	0	2 (0.1)	1.000
* speL*	0	0	1.000
* speM*	0	0	1.000
* Ssa*	2 (2.3)	2 (0.1)	0.012
* smeZ*	85 (98.8)	1,737 (99.4)	0.411
Other virulence factors			
*hasA* hyaluronic acid synthetase, capsule	86 (100)	1,03 (97.5)	0.266
Virulence-associated DNase, SDA1§	86 (100)	1,613 (92.3)	0.002
Patient characteristic			
Age, y			
<18	12 (14)	233 (13.3)	0.871
18–34	5 (5.8)	178 (10.2)	0.266
35–49	19 (22.1)	333 (19.1)	0.484
50–64	21 (24.4)	445 (25.5)	0.899
65–74	15 (17.4)	289 (16.5)	0.768
>75	14 (16.3)	269 (15.4)	0.762
Sex			
M	41 (47.7)	955 (54.7)	0.223
F	45 (52.3)	792 (45.3)	0.223
Clinical syndrome			
Cellulitis	25 (29.1)	633 (36.2)	0.205
Bacteremia without focus	11 (12.8)	276 (15.8)	0.544
Pneumonia	28 (32.6)	384 (22.0)	0.033
Necrotizing fasciitis	5 (5.8)	147 (8.4)	0.546
Streptococcal toxic shock syndrome	7 (8.1)	95 (5.4)	0.328
Death	19 (22.1)	260 (14.9)	0.089

*ABCs, Active Bacterial Core Surveillance System; iGAS, invasive group A *Streptococcus* disease. †By Fisher exact test. ‡All strain features, including antimicrobial susceptibility, pyrogenic exotoxin genes, and other virulence factors, were inferred from whole-genome sequencing data. §Detecting genetic marker for the *sda1* gene (GenBank accession no. AY452036.1), which is identical to the *sdaD2* gene (M5005_Spy1415; GenBank accession no. AAZ52033.1).

Patients infected by the M1_UK_ strain showed similar age, sex, and syndrome distribution compared with patients infected by non-M1_UK_ M1 GAS (Table), except that M1_UK_ isolates were more likely to be found in patients with pneumonia (33% vs. 22%; p = 0.033). The case-fatality rate was high for M1_UK_ iGAS infection (22%) although it was not significantly different from that of non-M1_UK_ M1 iGAS (15%; p = 0.089). In subgroup analysis stratified by time (2015–2018, 2019–2021) and location (GA, TN, and NY only), M1_UK_ isolates were associated with higher proportions of *speC*, *ssa*, *sda1*, and pneumonia compared with non-M1_UK_ isolates in all 3 subgroups (Appendix Tables 1‒3), except for *speC* in 2019–2021. The difference in subgroup analysis was generally not statistically significant, potentially caused by smaller sample size and reduced power in a subgroup.

## Conclusions

This study demonstrates a substantial increase of M1_UK_ lineage during 2019‒ 2021 in the ABCs sites in the United States. Additional data are needed to determine variance in M1_UK_ iGAS incidence across states outside the 10 states in ABCs. The proportion of M1_UK_ iGAS in ABCs remains much lower than that reported in England (*3*), Australia (*5*), and the Netherlands (*4*). We documented the mode of expansion for the M1_UK_ lineage in the United States by determining whether the 86 M1_UK_ iGAS cases could be explained by 1 recent introduction or multiple ones. We tracked the shape and characteristics of epidemiologic curves for each cluster, which could help understand different patterns of disease transmission. The increase was associated with the formation and expansion of multiple genomic clusters in which each cluster was mostly found in only 1 or 2 states. The results suggested that the M1_UK_ clone might have been introduced and circulated in different geographic locations in the United States, rather than spreading from a recent single introduction event.

In 2020, *emm*1 was the leading cause of iGAS only in Georgia and New York in ABCs. In that year, the proportion of M1_UK_ isolates among *emm1* iGAS was 38% (16/42) in Georgia and 25% (5/20) in New York. It appeared that M1_UK_ lineage followed the same state preferences as M1 in general. In recent years, there were rapidly expanding clusters of *emm* types that were not historically so prevalent within several different states, mostly pattern E lineages (*emm*11,49,82,92,60) and pattern D lineages (*emm*83,59,81), which was associated with increasing proportions of disadvantaged persons and led to drastic changes in *emm* type distributions in those states (*11**,**12*).

Although the *speC* and *ssa* genes were associated with M1_UK_ isolates, they were present in <5% of these isolates, and the biologic role of this association is unclear. It is crucial to monitor the spread of this variant and the associated virulence determinants to inform development of effective prevention and treatment strategies.

Appendix Additional information on expansion of invasive group A *Streptococcus* M1_UK_ lineage in active bacterial core surveillance, United States, 2019‒2021.
