# Supplementary material for: Expansion of Invasive Group A Streptococcus M1UK Lineage in Active Bacterial Core Surveillance, United States, 2019‒2021
Source: Emerg Infect Dis. 2023 Oct;29(10):2116–20. doi: 10.3201/eid2910.230675 (PMC10521608; doi:10.3201/eid2910.230675)
Supplement: Appendix — Additional information on expansion of invasive group A Streptococcus M1UK lineage in active bacterial core surveillance, United States, 2019‒2021. [file 23-0675-Techapp-s1.pdf]

Article DOI: https://doi.org/10.3201/eid2910.230675

EID cannot ensure accessibility for Supplemental Materials supplied by authors. Readers who have difficulty accessing supplementary content should contact the authors for assistance.

Expansion of Invasive Group A *Streptococcus* $M1_{UK}$ Lineage in Active Bacterial Core Surveillance, United States, 2019–2021

Appendix

Appendix Table 1. Strain and patient features of $M1_{UK}$ iGAS compared with other M1 iGAS in ABCs, USA, 2015–2018

Category	M1 iGAS		P-value[a]
	$M1_{UK}$ (N = 21)	Non-$M1_{UK}$ (N = 1209)	
Strain Feature[b]			
Antimicrobial Susceptibility			
Penicillin non-susceptible	0(0%)	0(0%)	1.000
Erythromycin non-susceptible	0(0%)	17(1.4%)	1.000
Clindamycin non-susceptible	0(0%)	16(1.3%)	1.000
Tetracycline non-susceptible	0(0%)	14(1.2%)	1.000
Levofloxacin non-susceptible	0(0%)	8(0.7%)	1.000
Pyrogenic Exotoxin Genes			
speA	21(100%)	1189(98.3%)	1.000
speC	3(14.3%)	11(0.9%)	0.001
speG	21(100%)	1209(100%)	1.000
speH, 27	0(0%)	1(0.1%)	1.000
speI	0(0%)	1(0.1%)	1.000
speJ	21(100%)	1209(100%)	1.000
speK	0(0%)	2(0.2%)	1.000
speL	0(0%)	0(0%)	1.000
speM	0(0%)	0(0%)	1.000
ssa	1(4.8%)	1(0.1%)	0.034
smeZ	21(100%)	1201(99.3%)	1.000
Other Virulence Factors			
hasA hyaluronic acid synthetase (CAPSULE)	21(100%)	1177(97.4%)	1.000
Active NADase mutation (NADASE_D330G)	21(100%)	1209(100%)	1.000
Pnga 3 - Clade 3 up-regulated promoter of the *nga* operon (PNGA3)	21(100%)	1209(100%)	1.000
Virulence associated DNase (SDA1) [c]	21(100%)	1119(92.6%)	0.394
Streptococcal inhibitor of complement (SIC)	21(100%)	1191(98.5%)	1.000
Patient Characteristic			
Age (yrs.)			
<18	5(23.8%)	167(13.8%)	0.200
18–34	1(4.8%)	115(9.5%)	0.713
35–49	4(19%)	230(19%)	1.000
50–64	2(9.5%)	313(25.9%)	0.127
65–74	5(23.8%)	200(16.5%)	0.375
> = 75	4(19%)	184(15.2%)	0.548
Male Sex	7(33.3%)	648(53.6%)	0.078
Clinical syndrome			
Cellulitis	6(28.6%)	444(36.7%)	0.502
Bacteremia without focus	2(9.5%)	188(15.6%)	0.759
Pneumonia	10(47.6%)	263(21.8%)	0.013

Category	M1 iGAS		P-value[a]
	M1UK (N = 21)	Non-M1UK (N = 1209)	
Necrotizing fasciitis	1(4.8%)	68(5.6%)	1.000
Streptococcal toxic shock syndrome	1(4.8%)	112(9.3%)	0.713
Death	5(23.8%)	188(15.6%)	0.357

[a] Fisher exact test
[b] All strain features, including antimicrobial susceptibility, pyrogenic exotoxin genes, and other virulence factors, were inferred from WGS data
[c] Detecting genetic target for the *sda1* gene (GenBank: AY452036.1) which is identical to the *sdaD2* gene (M5005_Spy1415; GenBank: AAZ52033.1)

Appendix Table 2. Strain and patient features of M1UK iGAS compared other M1 iGAS in ABCs, USA, 2019–2021

Category	M1 iGAS		P-value[a]
	M1UK (N = 65)	Non-M1UK (N = 538)	
Strain Feature[b]			
Antimicrobial Susceptibility			
Penicillin non-susceptible	0(0%)	0(0%)	1.000
Erythromycin non-susceptible	0(0%)	4(0.7%)	1.000
Clindamycin non-susceptible	0(0%)	4(0.7%)	1.000
Tetracycline non-susceptible	0(0%)	4(0.7%)	1.000
Levofloxacin non-susceptible	0(0%)	1(0.2%)	1.000
Pyrogenic Exotoxin Genes			
speA	65(100%)	531(98.7%)	1.000
speC	1(1.5%)	13(2.4%)	1.000
speG	65(100%)	538(100%)	1.000
speH, 27	0(0%)	0(0%)	1.000
speI	0(0%)	0(0%)	1.000
speJ	65(100%)	538(100%)	1.000
speK	0(0%)	0(0%)	1.000
speL	0(0%)	0(0%)	1.000
speM	0(0%)	0(0%)	1.000
Ssa	1(1.5%)	1(0.2%)	0.204
smeZ	64(98.5%)	536(99.6%)	0.290
Other Virulence Factors			
hasA hyaluronic acid synthetase (CAPSULE)	65(100%)	526(97.8%)	0.628
Active NADase mutation (NADASE_D330G)	65(100%)	538(100%)	1.000
Pnga 3 - Clade 3 up-regulated promoter of the *nga* operon (PNGA3)	65(100%)	538(100%)	1.000
Virulence associated DNase (SDA1) [c]	65(100%)	494(91.8%)	0.010
Streptococcal inhibitor of complement (SIC)	65(100%)	518(96.3%)	0.152
Patient Characteristic			
Age (yrs.)			
<18	7(10.8%)	66(12.3%)	0.842
18–34	4(6.2%)	63(11.7%)	0.214
35–49	15(23.1%)	103(19.1%)	0.507
50–64	19(29.2%)	132(24.5%)	0.449
65–74	10(15.4%)	89(16.5%)	1.000
> = 75	10(15.4%)	85(15.8%)	1.000
Male Sex	34(52.3%)	307(57.1%)	0.509
Clinical syndrome			
Cellulitis	19(29.2%)	189(35.1%)	0.408
Bacteremia without focus	9(13.8%)	88(16.4%)	0.722
Pneumonia	18(27.7%)	121(22.5%)	0.351
Necrotizing fasciitis	6(9.2%)	27(5%)	0.153
Streptococcal toxic shock syndrome	4(6.2%)	35(6.5%)	1.000
Death	14(21.5%)	72(13.4%)	0.090

[a] Fisher exact test
[b] All strain features, including antimicrobial susceptibility, pyrogenic exotoxin genes, and other virulence factors, were inferred from WGS data
[c] Detecting genetic marker for the *sda1* gene (GenBank: AY452036.1) which is identical to the *sdaD2* gene (M5005_Spy1415; GenBank: AAZ52033.1)

Appendix Table 3. Strain and patient features of $M1_{UK}$ iGAS compared with other M1 iGAS in ABCs, Georgia, Tennessee, and New York, USA, 2015–2021

Category	M1 iGAS		P-value[a]
	$M1_{UK}$ (N = 67)	Non-$M1_{UK}$ (N = 513)	
Strain Feature[b]			
Antimicrobial Susceptibility			
Penicillin non-susceptible	0(0%)	0(0%)	1.000
Erythromycin non-susceptible	0(0%)	3(0.6%)	1.000
Clindamycin non-susceptible	0(0%)	3(0.6%)	1.000
Tetracycline non-susceptible	0(0%)	4(0.8%)	1.000
Levofloxacin non-susceptible	0(0%)	3(0.6%)	1.000
Pyrogenic Exotoxin Genes			
speA	67(100%)	503(98.1%)	0.614
speC	2(3%)	6(1.2%)	0.234
speG	67(100%)	513(100%)	1.000
speH, 27	0(0%)	0(0%)	1.000
speI	0(0%)	0(0%)	1.000
speJ	67(100%)	513(100%)	1.000
speK	0(0%)	1(0.2%)	1.000
speL	0(0%)	0(0%)	1.000
speM	0(0%)	0(0%)	1.000
ssa	1(1.5%)	2(0.4%)	0.309
smeZ	66(98.5%)	511(99.6%)	0.309
Other Virulence Factors			
hasA hyaluronic acid synthetase (CAPSULE)	67(100%)	497(96.9%)	0.238
Active NADase mutation (NADASE_D330G)	67(100%)	513(100%)	1.000
Pnga 3 - Clade 3 up-regulated promoter of the *nga* operon (PNGA3)	67(100%)	513(100%)	1.000
Virulence associated DNase (SDA1) [c]	67(100%)	483(94.2%)	0.038
Streptococcal inhibitor of complement (SIC)	67(100%)	496(96.7%)	0.242
Patient Characteristic			
Age (yrs.)			
<18	7(10.4%)	65(12.7%)	0.698
18–34	3(4.5%)	56(10.9%)	0.131
35–49	14(20.9%)	86(16.8%)	0.392
50–64	20(29.9%)	141(27.5%)	0.666
65–74	11(16.4%)	95(18.5%)	0.740
> = 75	12(17.9%)	70(13.6%)	0.352
Male Sex	34(50.7%)	265(51.7%)	0.897
Clinical syndrome			
Cellulitis	20(29.9%)	209(40.7%)	0.110
Bacteremia without focus	10(14.9%)	73(14.2%)	0.854
Pneumonia	22(32.8%)	115(22.4%)	0.067
Necrotizing fasciitis	6(9%)	36(7%)	0.614
Streptococcal toxic shock syndrome	2(3%)	37(7.2%)	0.297
Death	16(23.9%)	91(17.7%)	0.241

[a] Fisher exact test
[b] All strain features, including antimicrobial susceptibility, pyrogenic exotoxin genes, and other virulence factors, were inferred from WGS data
[c] Detecting genetic marker for the *sda1* gene (GenBank: AY452036.1) which is identical to the *sdaD2* gene (M5005_Spy1415; GenBank: AAZ52033.1)

Appendix Table 4. National Center for Biotechnology Information (NCBI) accession numbers for 86 $M1_{UK}$ iGAS isolates in ABCs, USA, 2015–2021

LABID	Accession no.
20161865	SAMN07154227
20163230	SAMN08690692
20163247	SAMN08690707
20163759	SAMN08690786
20163766	SAMN08690793
20164844	SAMN08690990
20171243	SAMN08691789

LABID	Accession no.
20176277	SAMN08693085
20185573	SAMN14786371
20190915	SAMN14786372
20191426	SAMN14786373
20192677	SAMN27770449
20193256	SAMN27780161
20193583	SAMN27780320
20193886	SAMN27780470
20193887	SAMN27780471
20193893	SAMN27780477
20193898	SAMN27780481
20194013	SAMN27780516
20195248	SAMN27804123
20195260	SAMN27804134
20196014	SAMN27804283
20196019	SAMN27804288
20196384	SAMN27804385
20196388	SAMN27804389
20196611	SAMN27804478
20198369	SAMN27780982
20199063	SAMN27781018
20199200	SAMN27804941
20199375	SAMN27804995
20199539	SAMN27805044
20199542	SAMN27805047
20199544	SAMN27805049
20199547	SAMN27805052
20200042	SAMN27862100
20200355	SAMN27862225
20201194	SAMN27862470
20202264	SAMN27862703
20202637	SAMN27862824
20202640	SAMN27862827
20202644	SAMN27862831
20202645	SAMN27862832
20202887	SAMN27862860
20202901	SAMN27862874
20203218	SAMN27862963
20203717	SAMN27922172
20203724	SAMN27922179
20203731	SAMN27922448
20204044	SAMN27922453
20204050	SAMN27922459
20204249	SAMN27922205
20204602	SAMN27922519
20204643	SAMN27922291
20204721	SAMN27922551
20204976	SAMN27922611
20204977	SAMN27922612
20204990	SAMN27922625
20205195	SAMN27922637
20205200	SAMN27922642
20205206	SAMN27922648
20205208	SAMN27922650
20205291	SAMN27922665
20205577	SAMN27922747
20205702	SAMN27922765
20205803	SAMN27922774
20205806	SAMN27922777
20205809	SAMN27922780
20205888	SAMN27922832
20206013	SAMN27922854
20206239	SAMN27922912
20206275	SAMN27922927
20206370	SAMN27922966
20206744	SAMN27923064
20206746	SAMN27923066
20207010	SAMN27991921
20207017	SAMN27991927

LABID	Accession no.
20207373	SAMN27992014
20207374	SAMN27992015
20207375	SAMN27992016
20207376	SAMN27992017
20207377	SAMN27992018
20210265	SAMN27992206
20210348	SAMN27992224
20210486	SAMN27992278
20210513	SAMN27992289
20212820	SAMN27997101

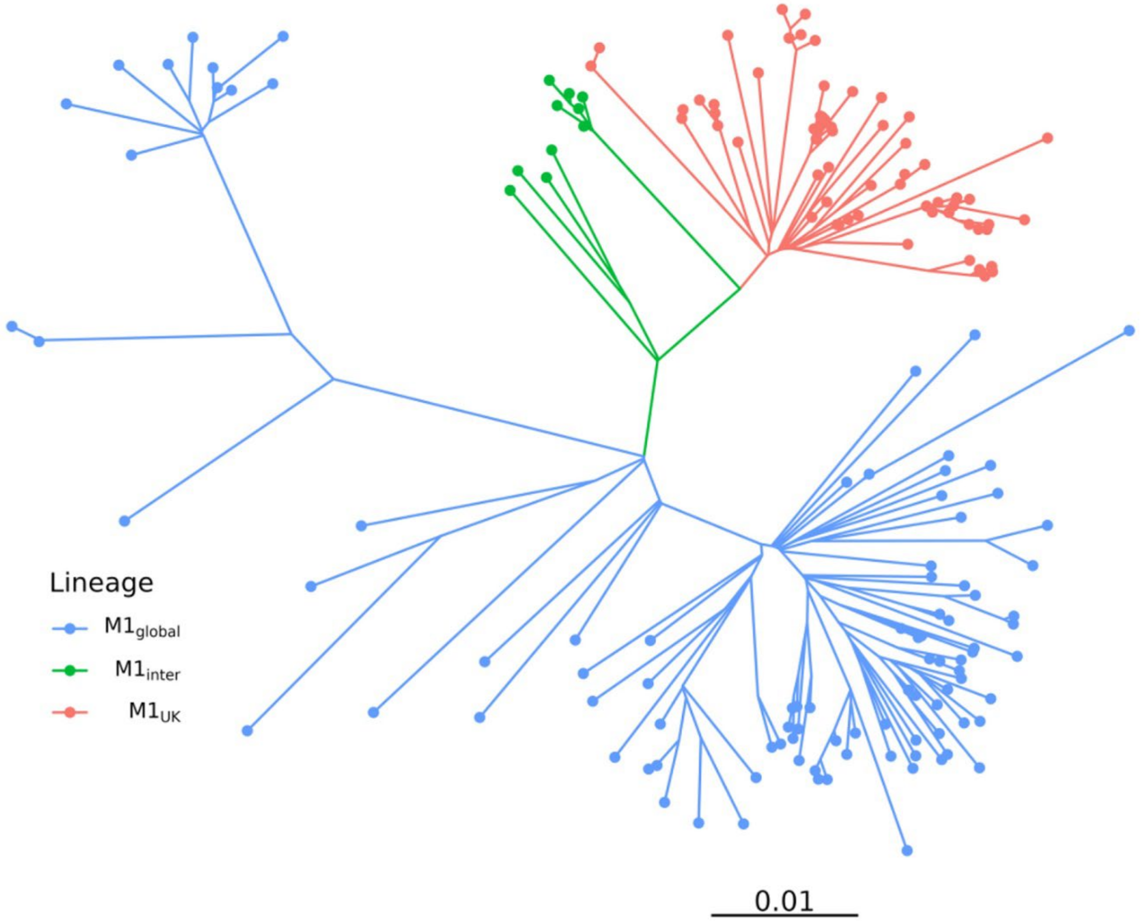

Appendix Figure 1. Core-genome phylogenetic tree of 86 M1UK isolates (red), 12 intermediate isolates (green), and 100 randomly selected M1Global isolates (blue) in the ABCs, 2015–2021. The tree is constructed based on 1657 core single-nucleotide variant sites by the kSNP3.0 software. Scale bar indicates expected nucleotide substitutions per site.

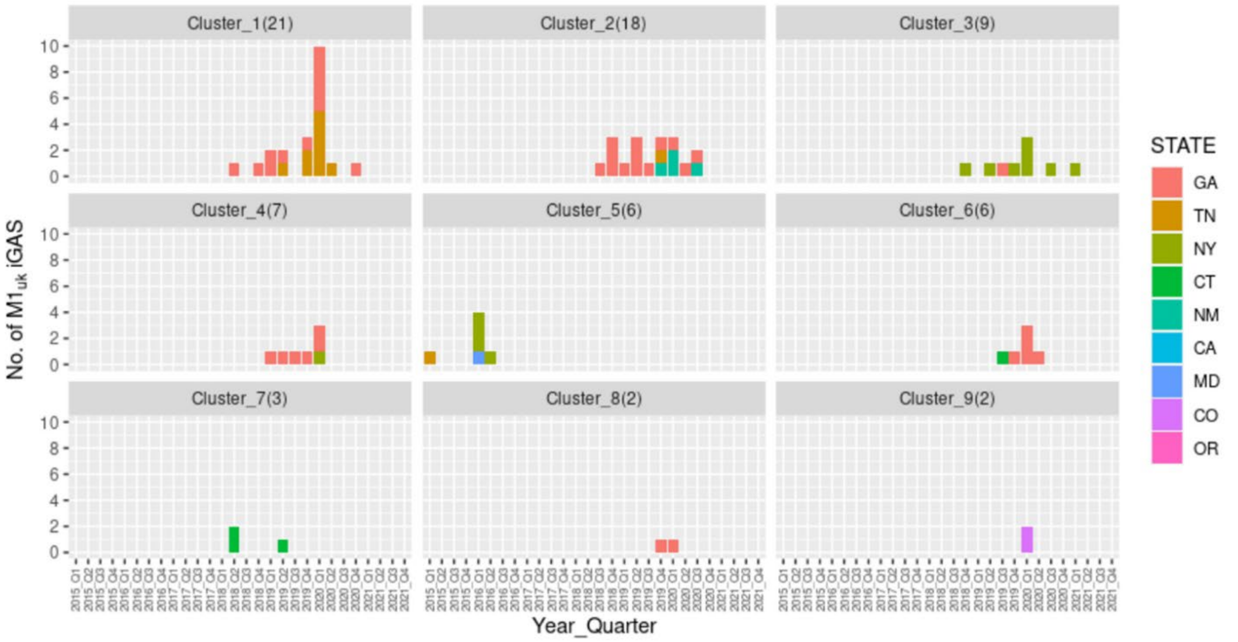

Appendix Figure 2. Number of cases over time for each of the 9 M1UK genomic clusters. Bar color indicates state. (CA = California, CO = Colorado, CT = Connecticut, GA = Georgia, MD = Maryland, NM = New Mexico, NY = New York, OR = Oregon, TN = Tennessee).
